# The cannabis paradox: contrasting role for marijuana in cardiovascular disease

**DOI:** 10.1038/s41392-022-01160-2

**Published:** 2022-09-05

**Authors:** Lin Deng, Bin Leng, Xiaowei Nie

**Affiliations:** 1 Department of Pharmacology, School of Medicine, Southern University of Science and Technology, Shenzhen, Guangdong 518055 China; 2 Department of Food Science and Technology, National University of Singapore, 2 Science Drive 2, Singapore, 117542 Singapore; 3 Shenzhen Key Laboratory of Respiratory Diseases, Shenzhen People’s Hospital (The First Affiliated Hospital, Southern University of Science and Technology), Shenzhen, 518055 China

**Keywords:** Cardiology, Drug screening

In a recent study published in *Cell*, Wei and colleagues describe the role of canabis in mediating inflammation and oxidative stress induced endothelial dysfunction and thereby contributing to the pathogenesis of atherosclerosis.^1^

Cardiovascular diseases (CVDs) are still the leading cause of death globally. There is an estimated 17.9 million people died from CVDs in 2019, representing 32% of all global deaths.^2^ Cannabis, also known as hemp or marijuana, is the most widely cultivated, trafficked and abused illicit drug worldwide especially in Western countries.

Medical cannabis have been used to treat a number of medical conditions including epilepsy, neuropathic pain, spasticity, chemotherapy related nausea and vomiting.^3^ Despite the benefical effects of cannabis, it is well known that cannabis use is strongly associated with CVDs such as atherosclerosis.^4^ The major psychoactive constituent in cannabis is ∆^9^-tetrahydrocannabinol (∆^9^-THC), which has shown selectively binding to cannabinoid receptor 1 (CB1/CNR1) in the endothelial cells (ECs) and is involved in the pathogenesis of CVDs.^1^ The underlying molecular mechanisms involved in the ∆^9^-THC induced vascular events are yet to be fully understood.

The authors first analyzed UK Biobank and found that cannabis was an independent predictor for myocardial infarction (MI). Further assessment found that inflammatory cytokines associated with the pathogenesis of atherosclerosis were significantly elevated after smoking a marijuana cigarette.^1^ In order to explore the molecular mechanisms, the authors aptly used ligand-based high-throughput virtual screening with the SWEETLEAD chemical database to identify novel selective CB1 antagonists. They found that genistein, a nature soybean flavonoid, is a neutral ligand for CB1 and can selectively bind to CB1.^1^ On the basis of this finding, the authors postulated that genistein can antagonize the effect of ∆^9^-THC induced endothelial dysfunctions.

**Fig. 1:**
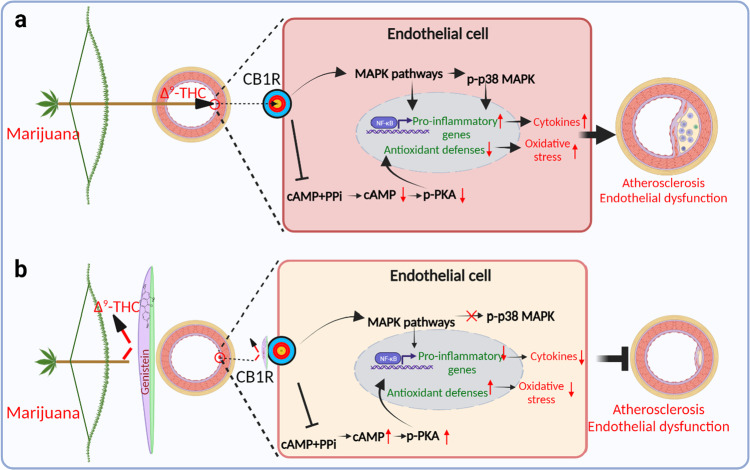
Protective effect of genistein on Δ9-THC-induced endothelial dysfunction in atherosclerosis. **a** △9-THC binds and activates the CB1R signaling, which activation causes inflammation and oxidative stress leading to endothelial dysfunctions in atherosclerosis through inducing PKA, p38 MAPK and NF-κB signaling pathways. **b** CB1R antagonist genistein alleviates the △9-THC-induced deleterious effects in endothelial cells, which suggest that genistein has promising therapeutic potential

Therefore, they first evaluated the cytotoxicity of ∆^9^-THC in various types of cells and found that ∆^9^-THC mainly induce cytotoxicity in human ECs. ∆^9^-THC exposure significantly induced inflammation-and ROS-related gene expression, and suppressed the antioxidant-related gene expression in different types of ECs. Human induced pluripotent stem cells (hiPSC-ECs) is an ideal platform for ex vivo models of vascular disease and screen toxicity and bioactive compounds.^1^ In addition, hiPSC-ECs are without environmental exposures, which can avoid the environmental effect on ∆^9^-THC induced phenotype in endothelial cells exposure to cannabis use. Interestingly, hiPSC-ECs only express the CB1 receptor. Thus, hiPSC-ECs were used to investigate the ∆^9^-THC induced cytotoxicity, inflammation, oxidative stress and the underlying molecular mechanisms. ∆^9^-THC treatment induced cellular oxidative stress, proinflammatory cytokines and chemokines production, increased monocyte adhesion and decreased the expression of antioxidant-related genes through activation of p38 mitogen-activated protein (MAP) kinase and NF-κB pathways in hiPSC-ECs.^1^ Following experiments show that genistein treatment attenuates ∆^9^-THC induced inflammatory, oxidative stress, monocyte adhesion, TLR4 expression and NF-κB phosphorylation in hiPSC-ECs (Fig. 1).

Various strategies including pharmacological inhibition, siRNA and CRISPR interference of CB1 have been applied to block CB1 in hiPSC-ECs. Genistein treatment significantly reversed ∆^9^-THC-induced inflammation-related genes and oxidative stress protective-related gene expression, which is consistent with the protective effect mediated by siRNA CB1 and CRISPR interference (CRISPRi).^1^ Vascular inflammation and oxidative stress are major triggers of endothelial dysfunctions contributing to the atherosclerosis initiation and progression. Genistein may serve as a potential therapeutic drug for treating ∆^9^-THC related atherosclerosis by reducing inflammation and oxidative stress. Multiple other cell types particitpate in atherosclerosis such as vascular smooth muscle cells and macrophages, which is important to access the role of genistein on these cells. To address the clinical and translational potential of genistein in atherosclerosis, the authors have set up an ex vivo wire myograph and two in vivo models of atherosclerosis (*Ldlr*^−/−^ and *Apoe*^−/−^ mice) to study the therapeutic effect of genistein in ∆^9^-THC induced atherosclerosis models. Genistein administration attenuates Δ^9^-THC mediated endothelial dysfunctions and pathological features of atherosclerosis including reducing atherosclerotic plaque formation and size, macrophage recruitment, neointimal thickening and fat deposition through inhibiting the NF-κB pathway.^1^

In addition, CB1 is the most abundant G-protein-coupled receptor (GPCR) in the mammalian brain, which mediate the psychoactive effects of marijuana. Psychiatric side effects need to be considered when developing the CB1 antagonist genistein as a therapeutic drug for atherosclerosis. Wei and colleagues demonstrated that genistein inhibits CB1 activity peripherally to prevent the proinflammatory, oxidative stress and pro-atherosclerotic effects of Δ^9^-THC while with lower CNS penetration due to the poor blood-brain barrier (BBB) penetration. Genistein preserved the beneficial central effects of Δ^9^-THC without psychiatric side effects in vivo,^1^ which suggest that CB1 antagonist genistein has clinically significant and would be promising therapeutics for atherosclerosis treatment. However, comparative analysis of different CB1 antagonist in various cell types involved in atherosclerosis both in vitro and in vivo can accelerate the clinical transition process. Collectively, the authors’ work represents the molecular mechanisms of Δ^9^-THC induced inflammation, oxidative stress and the therapeutic effects of genistein attenuating Δ^9^-THC-mediated endothelial dysfunctions in atherosclerosis.

Wei and colleagues comprehensively investigated the cannabis pro-inflammatory effects on ECs in atherosclerosis, which can be attenuated by CB1 antagonist genistein. This work may add important values for anti-inflammatory therapy in the treatment of atherosclerotic heart disease in the future. However, the therapeutic and the risks or side effects of cannabis use are still largely unknown. For exmaples, cannabis is widely used during cancer treatment. Recently study demonstrate that cannabis can suppress the antitumor immunity therapy through the inhibition JAK/STAT signaling pathway in T cells.^5^ The world is seeing an increased consumption of cannabis due to its legalization for recreational purposes. Therefore, the risk of non-medical use of cannabis should be further understood and educated public. As the inflammation and oxidative stress are one of the major contributors of many diseases including COVID-19, lung infection, cancer and autoimmunodisease. This study has opened the gate of future researches on the pharmacological and molecular mechanisms of cannabis in human diseases. It would be interested to evalute the medical and recreational use of cannabis and the underlying mechanisms in the inflammatory disorders and to develop specific therapeutic agents targeting cannabinoid receptors and endocannabinoids. Importantly, this research has rasied the public awareness on the health risks of marijuana use.
